# Mentalization Scale (MentS): Validity and reliability of the Iranian version in a sample of nonclinical adults

**DOI:** 10.1002/brb3.3114

**Published:** 2023-06-05

**Authors:** Ahmad Asgarizadeh, Elahe Vahidi, Parisa Sadat Seyed Mousavi, Ali Bagherzanjani, Saeed Ghanbari

**Affiliations:** ^1^ Faculty of Education and Psychology Shahid Beheshti University Tehran Iran; ^2^ Family Research Institute Shahid Beheshti University Tehran Iran; ^3^ Department of Clinical, Educational and Health Psychology University College London London UK

**Keywords:** mentalizing capacity, Mentalization Scale, psychometric properties

## Abstract

**Introduction:**

Mentalizing incapacity is increasingly identified as a common factor in psychopathology. The Mentalization Scale (MentS) is a cost‐effective measure built upon the dimensional model of mentalizing. We aimed to evaluate the psychometric properties of the Iranian version of MentS.

**Methods:**

Two samples of community‐based adults (*N*_1_ = 450, *N*_2_ = 445) completed different batteries of self‐report measures. In addition to MentS, participants completed measures of reflective functioning and attachment insecurities in the first sample and a measure of emotion dysregulation in the second sample.

**Results:**

Due to the conflicting results of confirmatory and exploratory factor analyses, an item‐parceling approach was applied, which replicated the original three‐factor structure of MentS, yielding Self‐Related Mentalization, Other‐Related Mentalization, and Motivation to Mentalize. The reliability and convergent validity of MentS were supported in both samples.

**Conclusion:**

Our findings provided preliminary evidence for using the Iranian version of MentS in nonclinical populations as a reliable and valid measure.

## Graphical abstract

The Mentalization Scale (MentS) is a relatively new theoretically‐grounded measure that assesses mentalizing capacity. The current study confirms the proposed three‐factor structure of the MentS in a sample of non‐clinical Iranian adults, as well as supporting its reliability and convergent validity.

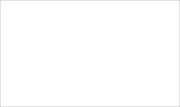

## 1 INTRODUCTION

Mentalizing is an imaginative capacity of humans, that is, envisioning one's and others’ behaviors with reference to the underlying mental states (e.g., Fonagy & Bateman, 2019). The concept of mentalization was primarily established by integrating two theories, namely, psychic reality development theory (Fonagy & Target, 1996; Target & Fonagy, 1996) and the social biofeedback theory of affect mirroring (Gergely & Watson, 1996). In their pioneering work, Fonagy et al. (2002) focused on the development of mentalizing capacity mainly in terms of parent–child interactions in the context of an attachment‐based relationship. However, their recent postulations (Fonagy et al., 2019; Luyten et al., 2020) interpose mentalizing capacity and attachment strategies between broader sociocultural context and resiliency. A growing body of literature is suggesting associations between mentalizing deficits and different categories of psychopathology, including personality disorders (Bateman et al., 2019; Drozek & Unruh, 2020; Fonagy & Luyten, 2016; Simonsen & Euler, 2019), anxiety disorders (Ballespí et al.,^2019^; Maresh & Andrews‐Hanna, 2021), eating disorders (Gagliardini et al., 2020; Robinson & Skårderud, 2019), and more recently, affective disorders (Luyten, Lemma, et al.,^2019^; Rifkin‐Zybutz et al., 2021). Nonetheless, nearly two decades after its foundation, the optimal way to assess this capacity is up for debate as new measures are still being introduced (e.g., Gagliardini & Colli, 2019; Müller, Wendt, & Zimmermann, 2021). Despite the emergence of various forms of measurements (i.e., self‐reports, interviews, experimental tasks, and performance‐based tasks), the Reflective Functioning Scale (RFS; Fonagy et al., 1998) is the gold standard. Nevertheless, RFS is applied to Adult Attachment Interview (AAI; George et al., 1996), which mainly aims at identifying attachment styles; ergo, some of the questions of AAI do not address mentalizing capacity (Taubner et al., 2013). Moreover, the feasibility of RFS is questionable: Administering RFS requires trained experts and a significant amount of time, and the final score—which is a single score ranging from −1 to 9—does not capture the heterogeneity of mentalizing deficits.

Considering RFS's limitations, several authors have developed cost‐effective self‐report measures for mentalizing capacity. The most reputable of these is the Reflective Functioning Questionnaire (RFQ; Fonagy et al., 2016), an eight‐item screening tool that assesses certainty (i.e., hypermentalizing) and uncertainty (i.e., hypomentalizing) about mental states. RFQ can discriminate between healthy and psychiatric clinical subjects, is significantly correlated with an array of psychopathologic features (e.g., self‐harm, symptomatic distress, and interpersonal problems), and has demonstrated acceptable internal consistency in psychiatric clinical populations (Badoud et al., 2015; Fonagy et al., 2016; Morandotti et al., 2018). However, a number of criticisms about RFQ have been raised since its development. Recently, using large psychiatric clinical and nonclinical samples, Müller, Wendt, Spitzer, et al.(2021) suggested that RFQ (1) measures mentalizing capacity as a unidimensional construct, (2) does not solely assess self‐related mentalizing capacity since the contents of its items also reflect emotional lability and impulsiveness, and (3) is unable to capture the pathologic aspect of certainty about mental states (i.e., hypermentalizing). The Mentalization Scale (MentS; Dimitrijević et al., 2018) is a relatively new theoretically grounded measure that assesses mentalizing capacity in three dimensions: Self‐Related Mentalization (MentS‐S), Other‐Related Mentalization (MentS‐O), and Motivation to Mentalize (MentS‐M). Similar to RFQ, MentS also differentiated between psychiatric clinical and nonclinical populations; was moderately to strongly correlated with measures of attachment styles, personality traits, and emotional intelligence; and showed acceptable internal consistencies (Dimitrijević et al., 2018). Furthermore, MentS scores were significantly higher in securely attached individuals compared with insecure groups (i.e., fearful, dismissing, and preoccupied). More recently, through its associations with RFS, Richter et al.(2021) found the construct validity of MentS to be satisfactory in a mixed psychiatric clinical sample. In this paper, we intended to evaluate the psychometric properties of the Iranian version of MentS.

### 1.1 Current study

We aimed to translate the MentS to Persian and validate the Iranian version of it in a sample of Iranian adults. Considering the inadequacy of measures to assess mentalizing in Persian‐speaking populations, validation of this theoretically grounded measure of mentalizing capacity is required for both research and clinical purposes. To this end, the first purpose of this study was to examine the factor structure of the MentS in a sample of Iranian adults. The second purpose of this study was to further explore the validity of the MentS by analyzing its associations with another measure of mentalizing capacity, attachment styles, and emotion dysregulation.

## 2 METHOD

### 2.1 Participants and procedure

Participants of this study were 895 adults (i.e., 18 years or older). They were randomly divided into two samples. In sample 1, participants were 450 adults (129 males and 321 females) with ages ranging from 18 to 65 (M = 27.70, SD = 8.52). In sample 2, participants were 445 adults (128 males and 317 females; age range = 18–43, M = 27.53, SD = 8.71). The sociodemographic characteristics of both samples are presented in Table 1. Sample 1 was used to examine the factorial structure of the MentS and to make the necessary modifications, if any.

**TABLE 1:** Sociodemographic characteristics of participants.

	Sample 1 (*n* = 450)		Sample 2 (*n* = 445)	
Characteristics	Frequency	Percentage	Frequency	Percentage
Sex				
Female	321	71.3	317	71.2
Male	129	28.7	128	28.8
Age				
18–24	236	52.5	189	42.5
25–34	141	31.3	175	39.3
35–44	56	12.5	57	12.8
45–54	11	2.4	18	4
55–65	6	1.3	6	1.3
Marital status				
Single	355	78.9	307	69
Married	95	21.1	138	31
Education level				
High school	3	0.7	16	3.6
Diploma	144	32	59	13.3
BSc	176	39.1	258	58
MSc	108	24	93	20.9
PhD	19	4.2	19	4.3

A Battery of self‐report measures was created using Porsline (https://porsline.ir). The URL consisting of the battery was distributed online via social media applications. Information regarding the purpose of the research and the confidentiality of data was provided, and all participants digitally signed the informed consent forms. All the procedures in the current study were approved by the ethics committee of the Attachment and Interpersonal Relationships Research Center, Shahid Beheshti University.

### 2.2 Measures

#### 2.2.1 Mentalization scale

The MentS (Dimitrijević et al., 2018) is a 28‐item scale consisting of three subscales: (1) Self‐Related Mentalization (MentS‐S), MentS‐O, and MentS‐M. The aforementioned subscales comprise 10, 8, and 10 items, respectively. MentS uses a five‐point Likert scale (from 1 = *completely incorrect* to 5 = *completely correct*), with higher scores suggesting a more sophisticated capacity for mentalizing. The reliability of MentS was tested in psychiatric clinical and nonclinical samples (Dimitrijević et al., 2018), which yielded satisfactory results (Cronbach's *α* = .75 and .84, respectively).

An Iranian version of the MentS was developed through a translation/back‐translation procedure. First, items of the MentS were translated from English to Persian by one of two authors that are fluent in English. Subsequently, a synthesis of the two translations was made. Then, the Persian version was back‐translated to English by another translator. Afterward, the back‐translated English version and the MentS were compared, and translation discrepancies were corrected.

#### 2.2.2 Reflective Functioning Questionnaire

The RFQ is a self‐report measure comprised of eight items that assess reflective functioning in a two‐dimension model: certainty (RFQc) and uncertainty (RFQu) about mental states. Participants rate items on a seven‐point Likert scale ranging from completely disagree to completely agree. Moderate agreements demonstrate an adaptive level of reflective functioning; extreme certainty about mental states, indicated by high RFQc scores, may represent hypermentalizing; and high scores in RFQu may reflect hypomentalizing. Internal consistencies for both RFQu and RFQc subscales have been demonstrated to be acceptable, with Cronbach's *α* values of .77 and .65, respectively (Fonagy et al., 2016). Furthermore, the test–retest reliability of RFQu and RFQc is found to be very good over 3 weeks (rs = .84 and .75; Fonagy et al., 2016). Reliability and construct validity of the two subscales of RFQ, and its Iranian version, are reported to be satisfactory in several studies (e.g., Morandotti et al., 2018; Seyed Mousavi et al., 2021).

#### 2.2.3 The Experiences in Close Relationships‐Revised Questionnaire

Attachment insecurities were measured using the Experiences in Close Relationships‐Revised (ECR‐R) Questionnaire (Fraley et al., 2000), which consists of 36 items that measure anxiety and avoidance in close relationships. Items are rated on a seven‐point scale from one (*strongly disagree*) to seven (*strongly agree*). In a meta‐analysis, Cronbach's *α* for anxiety and avoidance subscales were .90 and .91, respectively (Graham & Unterschute, 2015). Scores of ECR‐R were found to be temporally stable in a 6‐week time period (Sibley & Liu, 2004). The psychometric properties of the Iranian version of Difficulties in Emotion Regulation Scale (DERS) have also been found to be satisfactory (Nilforooshan et al., 2014).

#### 2.2.4 Difficulties in Emotion Regulation Scale

Emotion dysregulation was measured using the DERS (Gratz & Roemer, 2004), which assesses emotion dysregulation using discrete, albeit integrated, and components. DERS is a 36‐item scale comprising six subscales: (1) nonacceptance of emotional responses, (2) difficulties in engaging in goal‐directed behavior, (3) impulse control difficulties, (4) lack of emotional awareness, (5) limited access to emotion regulation strategies, and (6) lack of emotion clarity. Each of the six subscales comprises six items, with higher scores indicating elevated difficulties in regulating emotions. Participants can choose responses on a five‐point Likert scale, ranging from 1 = *almost never* (0%–10%) to 5 = *almost always* (91%–100%). The reliability and validity of the original version of DERS have been supported by several studies (e.g., Gratz & Roemer, 2004), as well as its Iranian version (Khanzadeh et al., 2012).

### 2.3 Data analysis

The factor structure of the Iranian version of MentS was explored in three steps. First, confirmatory factor analysis (CFA) was employed to examine the underlying three‐factor structure of the MentS. To this end, the Satorra–Bentler scaled *χ* ^2^ test was used to evaluate the overall fit of the factor model. As *χ* ^2^ is sensitive to sample size, model fit was also assessed using three additional fit indices, Root Mean Square Error of Approximation (RMSEA), Comparative Fit Index (CFI), and Goodness‐of‐Fit Index (GFI). Values greater than .90 for GFI and CFI and lower than .05 for RMSEA indicate good model fit (Hu & Bentler, 1999; MacCallum et al., 1996). An RMSEA value between .05 and .08 indicates a fair fit and between .08 and .10 demonstrates a mediocre fit (MacCallum et al., 1996).

Second, as the examined model did not fit the data, the analysis continuedusing Exploratory Factor Analysis (EFA). An essential aspect of the EFA is the determination of the reliable factors to be retained (Ledesma & Valero‐mora, 2007). Parallel analysis was used to determine the most accurate decision about the number of components (Hayton et al., 2004; O'connor, 2000).

Third, as results revealed a discrepancy between CFA and EFA (CFA was rejected, whereas EFA provided a reasonable solution), an item‐parceling approach was adopted. To this end, we used parcels as indicators of the latent variables. Parcels are combinations of items into small groups of items within scales or subscales and are used as construct indicators. According to Coffman and MacCallum (2005), this option offers several advantages. First, it reduces the number of parameters in the model, which more likely leads to less biased parameter estimates, achieving proper model solutions. In addition, using parcels reduces the influence of idiosyncratic features of the items (Bandalos & Finney, 2001). In this study, three parcels were considered for each latent variable, and items were randomly assigned to the parcels.

After establishing the factor structure of the Iranian version of MentS, we estimated the reliability by computing Cronbach's *α* for individual MentS subscales. The lowest acceptable *α* value can be regarded as .70 (Nunnally & Bernstein, 1994). Then, to further seek the validity of the MentS, associations among the three subscales of MentS, the dimensions of RFQ, emotion dysregulation, and attachment styles were examined using Pearson correlation analysis.

## 3 RESULTS

### 3.1 Factor structure of the MentS

Using sample 1, CFA suggested an unacceptable fit of the three‐factor model to the data (*χ* ^2^ (347) = 1063.68, *p* < .001, RMSEA = .07, 90% CI = [.07–.08], CFI = .74, GFI = .83). Examination of the modification indices, in order to find the sources of model misfit, showed many high values. Most of the modification indices suggested item cross‐loadings on another factor. The item with the highest modification index was deleted, and the analysis was conducted again. We repeated this procedure in an attempt to reach an acceptable fit. After the exclusion of four items (Items 26, 22, 25, and 27), although the *χ* ^2^ value was decreased, the fit of the model, as suggested by the alternative GFI, was still not acceptable (*χ* ^2^ (249) = 639.59, *p* < .001, RMSEA = .06, 90% CI = [.06–.07], CFI = .82, and GFI = .87).

Based on the abovementioned results, it was decided to shift to EFA. The MentS structure was examined by performing a principal component analysis (PCA) with an oblimin rotation. The PCA initially yielded six components with eigenvalues over 1, altogether explaining 52.63% of the variance. The final number of extracted components was determined by means of parallel analysis, which was performed using an SPSS syntax created by O'connor (2000). To identify the number of components with eigenvalues larger than those that might occur by chance, 1000 random datasets were created, each with 394 cases and 28 variables. In 95% of randomly created datasets, only the first three eigenvalues (1.46, 1.41, and 1.39) were notably lower than the corresponding ones in the original dataset (5.82, 2.96, and 2.05). Thus, the parallel analysis suggested a three‐component solution as the most fitting, with 38.70% of the variability explained. Individual item loadings on the retained components are listed in Table 2. Item loadings above .30 were used for factor interpretation. It is evident that the three extracted factors represent the three proposed subscales of the MentS (see Table 2). However, it should be noted that some items had a cross‐loading on another factor.

**TABLE 2:** MentS’ item loadings using EFA.

	Component		
Items	MentS‐M	MentS‐S	MentS‐O
1. I find it important to understand reasons for my behavior	**.59**	.214	.01
4. I often think about other people and their behavior	**.65**	−.15	−.09
7. When someone annoys me I try to understand why I react in that way	**.33**	−.06	−**.31**
9. I do not like to waste time trying to understand in detail other people's behavior	**.59**	.13	.26
13. I find it important to understand what happens in my relationships with people close to me	**.56**	−.01	−.23
15. To understand someone's behavior, we need to know her/his thoughts, wishes, and feelings	**.47**	−.02	−.22
16. I often talk about emotions with people that I am close to	**.36**	−.04	−.09
17. I like reading books and newspaper articles about psychological subjects	**.51**	−.01	−.08
24. I have always been interested in why people behave in certain ways	**.64**	−.06	−.05
27. Since we all depend on life circumstances, it is meaningless to think of other people's intentions or wishes	**.46**	**.33**	.13
8. When I get upset I am not sure whether I am sad, afraid, or angry	−.15	**.61**	−.12
11. Often I cannot explain, even to myself, why I did something	.01	**.69**	−.05
14. I do not want to find out something about myself that I will not like	**.31**	**.49**	.27
18. I find it difficult to admit to myself that I am sad, hurt, or afraid	.03	**.67**	.05
19. I do not like to think about my problems	.18	**.54**	.05
21. I am often confused about my exact feelings	−.19	**.54**	.05
22. It is difficult for me to find adequate words to express my feelings	−.27	**.64**	−**.39**
26. While people talk about their feelings and needs my thoughts often drift away	.11	**.51**	.01
2. When I make conclusions about other people's personality traits I carefully observe what they say and do	.25	.13	−**.40**
3. I can recognize other people's feelings	.21	.04	−**.61**
5. Usually I can recognize what makes people feel uneasy	.23	−.12	−**.60**
6. I can sympathize with other people's feelings	.14	.03	−**.55**
10. I can make good predictions of other people's behavior when I know their beliefs and feelings	−.02	.00	−**.63**
12. Sometimes I can understand someone's feelings before s/he tells me anything	.09	−.09	−**.65**
20. I can describe significant traits of people who are close to me with precision and in detail	−.04	.08	−**.54**
23. People tell me that I understand them and give them sound advice	.12	.08	−**.56**
25. I can easily describe what I feel	−.04	.23	−**.47**
28. One of the most important things that children should learn is to express their feelings and wishes	**.47**	.20	−**.37**

*Note*: Extraction method: principal component analysis; Rotation method: Oblimin with Kaiser normalization. Loadings set in boldface type indicate loadings higher than 0.3.

Abbreviations: MentS‐M, MentS‐motivation; MentS‐O, MentS‐others; MentS‐S, MentS‐self.

In light of the contradictory CFA and EFA findings, it was decided to adopt an item‐parceling approach. Application of the CFA procedure using parcels as indicators instead of individual items showed an acceptable fit in terms of GFI (*χ* ^2^ (24) = 87.19, *p* < .001, RMSEA = .07, 90% CI = [.06–.09], CFI = .94, GFI = .95). Similar results were obtained in sample 2 (*χ* ^2^ (24) = 98.77, *p* < .001, RMSEA = .07, 90% CI = [.06–.09], CFI = .95, GFI = .96). For both samples, parcel loadings were statistically significant yielding values ranging from .58 to .85 (see Table 3).

**TABLE 3:** Descriptive statistics and standardized factor‐loadings for parcels.

		Mean		SD		Standardized factor‐loadings	
Items^a^		Sample 1	Sample 2	Sample 1	Sample 2	Sample 1	Sample 2
S. P 1	11, 22	6.89	6.73	1.99	2.14	.72	.81
S. P 2	8, 14, 21	10.33	10.15	2.71	3.05	.85	.75
S. P 3	18, 19, 26	10.91	10.52	2.52	2.80	.69	.68
O. P 1	2, 6, 23, 25	16.21	16.60	2.33	2.36	.77	.81
O. P 2	3, 5, 12	11.36	11.29	2.04	2.18	.76	.70
O. P 3	10, 20, 28	12.31	11.63	1.74	2.12	.84	.60
M. P 1	1, 4, 13	12.69	12.43	1.88	1.92	.74	.78
M. P 2	7, 16, 27	11.26	11.35	2.01	2.13	.65	.58
M. P 3	9, 15, 17, 24	15.61	15.17	2.73	2.88	.67	.71

Abbreviations: M, MentS‐motivation; O, MentS‐others; P, parcel; S, MentS‐self; SD, standard deviation.

^a^ Reflects the items which belong to the parcel.

Cronbach's *α* coefficients for MentS‐S, MentS‐O, and MentS‐M were respectively .79, .78, and .75 in the first sample, as well as .82, .83, and .73 in the second sample. These values indicate satisfactory internal consistency for research purposes (Nunnally & Bernstein, 1994). The intercorrelations among the three subscales, as well as descriptive statistics for them, are presented in Table 4.

**TABLE 4:** Descriptive statistics and Pearson correlation coefficients for MentS.

	Pearson correlations			Mean		SD	
	MentS‐S	MentS‐O	MentS‐M	Sample 1	Sample 2	Sample 1	Sample 2
MentS‐S	1	.42**	.35**	28.13	27.58	6.06	6.77
MentS‐O	.30**	1	.65**	39.88	39.52	5.00	5.75
MentS‐M	.32**	.52**	1	39.56	38.94	5.36	5.59

*Note*: Values below the diagonal refer to sample 1, and values above refer to sample 2.

Abbreviations: MentS‐M, MentS‐motivation; MentS‐O, MentS‐others; MentS‐S, MentS‐Self; SD, Standard Deviation.

^**^ *p* < .01.

### 3.2 Associations with related measures

To provide further validity evidence based on relations with other variables, we calculated Pearson correlations among the MentS subscales, reflective functioning, emotion dysregulation, and attachment styles (see Table 5). The obtained correlations for all three subscales of MentS were in the expected direction and all statistically significant, with the exception that MentS‐M was not associated with RFQ‐Uncertainty.

**TABLE 5:** Pearson correlations between MentS subscales and reflective functioning, and attachment insecurities.

Measures	RFQ‐Certainty	RFQ‐Uncertainty	ECR‐R‐avoidance	ECR‐R‐anxiety	DERS
MentS‐S	.50**	−.38**	−.44**	−.45**	−.65**
MentS‐O	.36**	−.14**	−.22**	−.27**	−.33**
MentS‐M	.22**	−.03	−.13**	−.16**	−.21**

Abbreviations: DERS, Difficulties in Emotion Regulation Scale; ECR‐R, Experiences in Close Relationships—Revised Questionnaire; MentS‐M, MentS‐motivation; MentS‐O, MentS‐others; MentS‐S, MentS‐self; RFQ, Reflective Functioning Questionnaire.

^**^ *p* < .01.

## 4 DISCUSSION

The present study aimed to validate the Iranian version of MentS in a sample of nonclinical adults. MentS is a theoretically grounded measure of mentalizing capacity that assesses this capacity based on a three‐factor model consisting of Self‐Related mentalization, MentS‐O, and MentS‐M. The purposes of this study were to examine the factor structure of the MentS and to provide further evidence for its validity by investigating its associations with theoretically related measures. The main findings are summarized below.

### 4.1 Factor structure of the MentS

First, we examined the factor structure of the MentS using CFA in sample 1. Findings revealed an unacceptable fit to the hypothesized three‐factor model. A possible explanation for why in several instances CFA could not reach a satisfactory fit is offered by Marsh et al. (2014). They discuss that the hypothesis of zero cross‐loadings in CFA is a stringent assumption in the field of social sciences. This issue is perhaps enlarged in the case of MentS, in which the large number of items of subscales facilitates their cross‐loading.

On the other hand, EFA and CFA, using an item‐parceling approach, supported the three‐factor structure of the MentS. The hypothesized structure was supported in EFA, where items are free to load on any factor. Also, when using an item‐parceling approach, CFA supported the three‐factor structure of the MentS. Due to the aggregation of item ratings, the item‐parceling approach has the advantage of reducing noise unrelated to the construct and accordingly increases the ratio of the true score to the total score (Matsunaga, 2008).

Besides, the Cronbach's *α* for all three subscales in our study were shown to be satisfying. However, in our sample, MentS‐M yielded the lowest internal consistency, which is in concordance with the other studies (Dimitrijević et al., 2018; Richter et al.,^2021^).

### 4.2 Associations with related measures

In order to provide further evidence for the validity of the Iranian version of MentS, we investigated its correlations with measures of reflective functioning, attachment insecurities, and emotion dysregulation. Except for the correlation between MentS‐M and RFQu, all of the correlations between subscales of MentS and subscales of RFQ were significant and in the expected direction, which provides further evidence for the validity of the MentS.

All three subscales of the MentS were positively associated with RFQc, and MentS‐S and MentS‐O were negatively associated with RFQu. These associations were in the expected direction, as in the previous studies RFQc and RFQu were, respectively, positively and negatively associated with constructs related to mentalization (e.g., empathy and mindfulness; Badoud et al., 2015; Fonagy et al., 2016). Believing in the opacity of the mental states in self and others’ minds, that is, not having an inflated sense of certainty nor getting stuck in a general state of uncertainty, can be considered a critical element of the mentalizing process. Since mental states are opaque, being tentative about our inferences and taking an inquisitive stance are necessary for effective mentalizing (Fonagy & Bateman, 2019). The patterns of correlations between subscales of the MentS and the DERS, which indicated a negative association between mentalizing capacity and emotion dysregulation, provided further support for the validity of the MentS, as well. A vast amount of theoretical literature has discussed the association between mentalization and emotion regulation (Fonagy et al., 2002; Greenberg et al., 2017), and some empirical studies have provided evidence for their association (e.g., Vahidi et al., 2021).

All three subscales of MentS were negatively associated with attachment anxiety and attachment avoidance. These findings were, for the most part, in line with the suggestions of the original study (Dimitrijević et al., 2018) and can provide further evidence for the validity of the MentS. It is suggested that anxiously attached individuals could be characterized by an unstable capacity to mentalize, disposing them to emotion dysregulation (Mayes, 2000; Nolte et al., 2013; Vrticka & Vuilleumier, 2012; Vrtička et al., 2008). Moreover, individuals with avoidant attachment style may lack the capacity for genuine mentalizing: in highly arousing situations, they are prone to hypermentalizing (i.e., the tendency to overinterpret mental states beyond the available evidence) and regressing to pretend mode of functioning (Luyten, Malcorps, et al.,^2019^).

Our findings should also be considered in light of their cultural context. For instance, comparing the results of Serbian (Dimitrijević et al., 2018), Polish (Jańczak, 2021), Chinese (Wen et al., 2022), and Iranian versions suggest that culture may be a moderator in the association between attachment avoidance and mentalizing the self. The influence of cultural background on attachment, particularly attachment avoidance, has been corroborated (Friedman et al., 2010). However, the abovesaid moderation cannot be facilely attributed to the difference between collectivistic–individualistic cultures (i.e., lower levels of self‐focused mentalizing in collectivistic cultures; Aival‐Naveh et al., 2019), as the association between avoidance and self‐focused mentalizing varies significantly between the collectivistic cultures of China, Serbia, and Iran. Hence, cultural complexities of mentalizing capacity, as measured by MentS, shall be acknowledged and further studied.

### 4.3 Limitations and suggestions for future research

Several limitations of the current study and suggestions for future researchers should be mentioned. First, our research design may not warrant the generalizability of the findings since no specific sampling technique was followed. Research with representative samples could provide more information on the psychometric properties of the MentS. Second, in examining validity issues, this study is limited since it only used self‐report measures. Future researchers are suggested to judge the validity of the Iranian version of the MentS against other instruments (e.g., interview‐based scales such as the RFS). Moreover, although the correlation coefficients between MentS and related variables were statistically significant, they were relatively low; thus, our findings should be interpreted cautiously. Third, the next steps in the validation of the Iranian version of MentS should consider its psychometric properties in psychiatric clinical samples.

## 5 CONCLUSION

In conclusion, this study confirms the proposed three‐factor structure of the MentS in a sample of nonclinical Iranian adults. Associations between subscales of MentS and RFQ, attachment insecurities, and emotion dysregulation provided further support for the validity of the Iranian version of the MentS. To the extent we know, this is the first study to investigate the psychometric properties of the MentS in Iran. The findings of this study also add to the previous literature on the cross‐cultural assessment of mentalizing capacity, specifically using MentS.

## CONFLICT OF INTEREST STATEMENT

The authors declare that they have no conflict of interest.

## FUNDING INFORMATION

The authors have no funding to disclose.

### PEER REVIEW

The peer review history for this article is available at https://publons.com/publon/10.1002/brb3.3114.

## Data Availability

The data that supports the findings of this study are available from the corresponding author upon reasonable request.
